# Competitive Bio-Accumulation Between Ammonia and Nitrite Results in Their Antagonistic Toxicity to *Hypophthalmichthys molitrix*: Antioxidant and Immune Responses and Metabolic Detoxification Evidence

**DOI:** 10.3390/antiox14040453

**Published:** 2025-04-10

**Authors:** Honghui Guo, Yiwen Li, Heng Ge, Hang Sha, Xiangzhong Luo, Guiwei Zou, Hongwei Liang

**Affiliations:** 1 Yangtze River Fisheries Research Institute, Chinese Academy of Fisheries, Wuhan 430223, China; guohonghui@yfi.ac.cn (H.G.); meetmitchell@163.com (Y.L.); hg1154483105@163.com (H.G.); sh1812@yfi.ac.cn (H.S.); lxz@yfi.ac.cn (X.L.); zougw@yfi.ac.cn (G.Z.); 2 College of Fisheries, Huazhong Agricultural University, Wuhan 430070, China

**Keywords:** ammonia, nitrite, liver, spleen, antagonistic effect, silver carp

## Abstract

Ammonia and nitrite, as major aquatic pollutants, exhibit significant toxicity toward aquatic organisms. However, their interactive effects on fish are unclear. Aiming to determine their interactive effects, silver carp (*Hypophthalmichthys molitrix*) were exposed to ammonia, nitrite or ammonia + nitrite for 72 h. Silver carp exhibited pathological damage in the liver and spleen and significant increases in MDA, SOD and CAT in the liver and plasma after ammonia or nitrite exposure. Thus, ammonia and nitrite caused significant histology damage through inducing oxidative stress, and the antioxidative response of SOD−CAT was initiated by silver carp to defend them. A transcriptomic analysis suggested that disruptions in immune responses and metabolism were the main toxic effects caused by ammonia and nitrite. Specifically, nitrite decreased splenic TNF-α and IL-1β but increased splenic C4. Ammonia decreased splenic TNF-α and C4 but increased splenic IL-1β. We noted significant interactions between ammonia and nitrite, and the pathological changes and IBR in the co-exposure groups were less severe than those in the single-factor exposure groups, indicating that ammonia and nitrite have an antagonistic effect. Significant decreases in plasmatic ammonia and NO_2_^−^+NO_3_^−^ were induced by nitrite and ammonia, respectively. Moreover, the plasmatic glutamine, urea-N, and glutamine synthetase and glutamate dehydrogenase activities increased significantly under ammonia and nitrite exposure, while T-NOS decreased significantly. These results suggest an antagonistic interaction between ammonia and nitrite in silver carp, possibly resulting from competitive bioaccumulation. Consequently, the simultaneous monitoring and control of both ammonia and nitrite concentrations are essential to mitigate their compounded toxic effects, which might be exacerbated under isolated exposure conditions.

## 1. Introduction

Ammonia and nitrite, which are persistent pollutants in aquatic environments, are highly toxic to aquatic organisms and affect human food safety. Ammonia includes NH_3_ and NH_4_^+^ in water, and NH_3_ is more poisonous to aquatic life due to its lipid solubility, which leads it to diffuse across gill membranes. The World Health Organization (WHO) recommends an ammonia level in drinking water of 0.05–3 mg/L (NH_4_^+^). However, ammonia levels can reach >20 mg/L if sewage treatment is ineffective or nonexistent [1]. In aquaculture, ammonia has been reported at levels of up to 46.11 mg/L [2]. Similarly, nitrite (NO_2_^−^) level can reach up to 20 mg/L in intensive aquaculture systems [3]. Ammonia and nitrite commonly appear concurrently as nitrite is formed from ammonia by nitrification processes. In the same aquaculture wastewater, the ammonia and nitrite levels reached 11.44 mg/L and 9.86 mg/L, respectively [4]. Therefore, concerns have been raised regarding nitrite and ammonia accumulation and their synergistic effects in aquatic environments [5].

Ammonia causes convulsions and death in fish due to the excessive activation of N-methyl-D-aspartate receptors and subsequent cell death in the central nervous system [6]. Recently, ammonia toxicity has been documented to interfere with amino acid transport and disrupt the antioxidant and immune capacity [7,8]. One toxic effect of nitrite is methemoglobinemia as hemoglobin is oxidized by NO_2_^−^, i.e., the ferrous ion (FeII) is oxidized to ferric ion (FeIII) and is unable to bind to and carry molecules of oxygen [9]. Oxidative stress due to high levels of reactive oxygen species (ROS) is also induced by nitrite, which disturbs various physiological functions and even results in death [10,11]. Moreover, ammonia and nitrite’s toxicity varies with the presence of other pollutants. Significant interactions between ammonia and microcystin regarding superoxide dismutase (SOD) and malondialdehyde (MDA) were observed in bighead carp [12]. In addition, ammonia’s toxicity can be enhanced by oxygen and is affected by salinity in aquatic animals [13,14]. Similarly, nitrite’s toxicity is affected by environmental factors such as the temperature, sodium chloride, and pH [15,16,17]. Although the toxicity of ammonia or nitrite alone has been reported, reports on their combined effects on fish are limited. Nitrite enhanced the ammonia-induced increases in nitrogen metabolism and the production of urea-N and other types of organic-N in *Marsupenaeus japonicus* [18]. Similarly, the combined exposure of *M. japonicus* to ammonia and nitrite induced synergistic and simultaneous effects on respiratory parameters, the acid–base balance and osmoregulation [5]. Lin et al. (2024) reported that combined stress due to ammonia and nitrite caused more extensive histopathological damage and oxidative stress in *Litopenaeus vannamei* [19]. There were significant synergistic effects of ammonia and nitrite on alanine aminotransferase and aspartate aminotransferase in *L. vannamei* [20]. Ammonia significantly enhanced the Cu/Zn superoxide dismutase (Cu/Zn-SOD) activity in *Macrobrachium rosenbergii* by interacting with nitrite [21]. However, an antagonistic effect of ammonia and nitrite on the LC50 value and gill damage was observed in *Macrobrachium amazonicum*, which had greater tolerance to ammonia and nitrite concentrations when subjected to these compounds combined rather than alone [22]. Moreover, the combined effect of ammonia and nitrite on *Penaeus monodon* post-larvae was antagonistic at 48 h and 72 h of exposure but synergistic after 96 h of exposure based on the LC50 values [23]. Their antagonistic effects might be explained by the interaction of nitrite with some chloride ions, such as ammonium chloride, decreasing its toxic effect through the binding of chloride free radicals and causing the compounds to enter a conformation that is difficult to absorb via the gills [22]. Moreover, the toxic effects of nitrite and ammonia exposure may vary depending on differences in shrimp species. Notably, there are limited reports of their combined effects on fish. Recently, we reported a time-dependent interactive effect of nitrite and ammonia on the inflammatory and immune responses in the head kidneys of silver carp (*H. molitrix*) [24]; however, the underlying mechanism is unclear.

The objective of the present study was to evaluate the combined effects of ammonia and nitrite on fish. Silver carp, a filter-feeding fish, has been expanded to over 88 countries as it can improve the water quality by controlling algal blooms [25]. In 2020, its worldwide yield was 4.897 million tonnes [26]. Thus, in the present study, silver carp was chosen and immersed in ammonia, nitrite and a combination of the two for 72 h; then, plasma biochemical parameters, oxidative stress, the immune response and the histological structure were examined to evaluate the combined effects of ammonia and nitrite. We also used the RNA-seq technique (RNA-seq) to identify the underlying genes and pathways related to combined stress, thus providing new knowledge about the molecular mechanisms of ammonia and nitrite toxicity.

## 2. Materials and Methods

### 2.1. Chemicals

Ammonium chloride power (NH_4_Cl, 99.5% purity) and sodium nitrite power (NaNO_2_, 99.5%) were used according to previous studies on the combined exposure of ammonia and nitrite [5,19,20]. All other reagents used in this study were of analytical grade.

### 2.2. Silver Carp Maintenance and the Exposure Protocol

Silver carp (94.21 ± 4.88 g) were obtained from the Yangtze River Fisheries Research Institute in Jingzhou city, Hubei province, China. Before the experiment, they were maintained in a recirculating freshwater system (200 L) and acclimatized to the laboratory conditions for two weeks. The temperature was 25 °C, the dissolved oxygen (DO) was maintained at >6.0 mg/L and the pH was 8.2. During acclimation, the fish were kept with no food. A natural photoperiod was maintained throughout the trial. After the acclimation period, silver carp (n = 180) were assigned randomly into 12 glass tanks at a density of 15 fish per tank. The 12 tanks were randomly divided into 4 groups that contained different combinations of ammonia (15 mg/L) and nitrite (10 mg/L) for 72 h of exposure, in accordance with previous studies [27,28,29,30]. Ammonia and nitrite levels were used in this study in accordance with the 96 h LC50 values of ammonia (29.66 mg/L) and nitrite (19.51 mg/L) in silver carp and the environmental levels reported by previous studies [4,31]. The experiment followed a fully factorial design, i.e., there were four treatment combinations contained in three replicate tanks. Stock solutions were prepared by separately dissolving NaNO_2_ (15 g) and NH_4_Cl (29.72 g) in 1 L of distilled water each. The stock solution was used to adjust the experimental levels of nitrite and ammonia in each exposure group. The concentrations of ammonia and nitrite were measured using Nessler’s reagent colorimetric method and the N-(1-Naphthyl) ethylenediamine dihydrochloride spectrophotometric method, respectively. The levels of ammonia and nitrite were maintained by adding stock solutions of NH_4_Cl and NaNO_2_ every day. The concentration of NH_3_ was calculated based on water temperature, pH and total ammonia conditions. During the experiment, the temperature was 25 °C, the pH was 8.2 ± 0.06 and the DO was above 5.0 mg/L. Dead fish were removed immediately from tanks.

After 24 h, 48 h and 72 h of ammonia and nitrite exposure, 12 individuals were randomly sampled from each treatment group and anesthetized using a 0.02% MS-222 solution. Blood was sampled from the caudal vein using heparinized syringes, and then plasma was separated by centrifugation for biochemical parameter determination. Livers and spleens from three individuals in each group were dissected out and fixed using 10% neutral buffered formalin. Samples of livers and spleens from other fish were excised immediately, frozen in liquid nitrogen and then stored at −80 °C for the analysis of oxidation, antioxidation, immune factors and gene expression. The experiment was approved by the Animal Experimental Ethical Inspection of Laboratory Animal Centre, Yangtze River Fisheries Research Institute, Chinese Academy of Fishery Sciences (protocol code: 2022-GHH-01), and followed the institutional guidelines for animal experiments.

### 2.3. Detection of Physiological and Biochemical Parameters in Plasma

Blood samples were centrifuged (845× *g*, 20 min, 4 °C) to preparate plasmatic samples. Each experimental group had six replicates and one replicate included the plasma from two individuals from the same tank. Levels of ammonia (NH_4_^+^+NH_3_), nitrite (NO_2_^−^) + nitrate (NO_3_^−^), Cl^−^, Na^+^, MDA, SOD, catalase (CAT), glutathione peroxidase (GPx), total nitric oxide synthase (T-NOS), glutamate (Glu), urea-N, glutamine (Gln), glutamate dehydrogenase (GDH), glutamine synthetase (GS) and total protein were determined using commercial kits from the Nanjing Jiancheng Bioengineering Co. (Nanjing, China).

### 2.4. Detection of Oxidative and Antioxidative Parameters in the Liver

Liver samples were homogenized (1:10, *w*/*v*) in a cold (4 °C) saline solution (0.85% NaCl) and centrifuged at 845× *g* for 15 min at 4 °C. Supernatants were used as the enzyme source. The MDA content and the activities of SOD, CAT and GPx were determined using commercial kits (Nanjing Jiancheng Bioengineering Institute, Nanjing, China). All experiments were conducted using three biological replicates.

### 2.5. Detection of Immune Parameters in the Spleen

Spleen samples from three fishes of same group were homogenized in 0.85% sodium chloride, then centrifuged at 845× *g* for 15 min (4 °C) to collect the supernatant. Each experimental group had three replicates. Supernatants were used to test the concentrations of immunoglobulin M (IgM), interleukin 1β (IL-1β), tumor necrosis factor α (TNF-α), C3, C4, lysozyme and total protein using commercial kits purchased from the Jiancheng Bioengineering Institute (Nanjing, China).

### 2.6. Histological Evaluation

Histological examinations of liver and spleen were performed according to a previous study [32]. Liver and spleen samples (3–5 mm^3^) were fixed in 10% neutral-buffered formalin for 48 h. Then, tissues were dehydrated in a graded ethanol series of 70–100%. Thereafter, they were hyalinized in a mixture of xylene and ethanol (*v*/*v*, 1:1) for 10 min and then in 100% xylene for 10 min. After immersion in paraffin for 60 min at 58 °C, the liver and spleen samples were embedded, sectioned (5 μm) and stained with Hematoxylin and Eosin (H&E). Histopathological assessment was carried out under a light microscope (Nikon H600L Microscope and image analysis system, Tokyo, Japan). Histological changes were further evaluated quantitatively according to the method reported by Corbett et al. [33]. Images of liver and spleen sections were captured at 400× magnification. Three images per tissue section were randomly selected for quantification analysis according to area coverage or lesion frequency. A severity score value from 0 to 6 was assigned for the degree and extent of each alteration: 0—unchanged; 1 or 2—mild; 3 or 4—moderate; 5 or 6—severe. The detailed analysis protocol is supplied in the Supporting Information (Supplementary Text S1).

### 2.7. Total RNA Extraction and Transcriptome Sequencing

After 48 h of ammonia and nitrite exposure, the total RNA was extracted from the liver and spleen using the TRIzol reagent (Invitrogen, Waltham, MA, USA) according to the supplier’s guidelines. The integrity and quality of the RNA were tested on a Bioanalyzer 2100 (Agilent Technologies, Santa Clara, CA, USA) and using 1% agarose gel electrophoresis, respectively. The RNA concentrations were determined using a NanoDrop instrument (Thermo Scientific, Waltham, MA, USA). RNA (122 μg) was used to prepare the RNA-seq libraries, employing a TruSeq™ RNA Sample Preparation Kit (Illumina, San Diego, CA, USA). Library sequencing was accomplished using paired-end sequencing on the Illumina HiSeq 2500 sequencing platform. The sequencing data can be accessed at the NCBI SRA database (CRA015776). Shanghai Paisennuo Biotechnology Co. Ltd. (Shanghai, China) performed the bioinformatic analysis of the RNA-Seq data bioinformatically. Identified differentially expressed genes (DEG) were subjected to gene ontology (GO) analysis and Kyoto Encyclopedia of Genes and Genomes (KEGG) enrichment analysis in the KOBAS software (version 2.1.1). The detailed analysis protocol is supplied in the Supporting Information (Supplementary Text S2).

### 2.8. Real-Time Quantitative Real-Time Reverse Transcription PCR (qRT-PCR)

The gene-specific primers listed in Table S1 were designed according to the sequences obtained from the full-length transcriptome sequencing of silver carp. Total RNA (1 μg) was reverse-transcribed to cDNA using a FastKing RT Kit (TianGen, Beijing, China). Next, the RT-qPCR reactions were performed with 5 μL of 2×SYBR Green MasterMix reagent (Takara, Shiga, Japan), 1 μL of diluted (1:10) cDNA, 0.4 μL of forward and reverse primers (10 μM) and 3.6 μL of H_2_Ovon in a CFX96 Multicolor Real-Time PCR Detection System (Bio-Rad, Hercules, CA, USA). The thermal cycling profile consisted of an initial denaturation step at 95 °C for 20 s, followed by 40 cycles of denaturation at 95 °C for 15 s, annealing at the melting temperature (TM) for 20 s and extension at 72 °C for 20 s. All samples were processed in triplicate, and the data were normalized against the expression of *β-actin* [34]. The expression level of the genes was calculated using the 2^−ΔΔCt^ method.

### 2.9. Statistical Analysis

All statistical analyses were performed using two-way (ammonia and nitrite) analysis of variance (ANOVA) followed by Dunnett’s multiple range test in SPSS 20.00 for Windows (IBM, Corp., Armonk, NY, USA). Data were plotted using GraphPad Prism 6 software (GraphPad Inc., San Diego, CA, USA). The data were subjected to integrated biomarker response (IBR) analysis, which integrates all of the measured biomarker responses into an integrative index to assess stress levels [35]. High IBR values reflected the enhanced biological responses and poor health condition of the organisms. Detailed information on the calculation procedure is provided in the Supporting Information (Supplementary Text S3). Differences were measured and were considered to be statistically different at *p* < 0.05.

## 3. Results

### 3.1. Hepatic and Splenetic Histopathology

**Figure 1:**
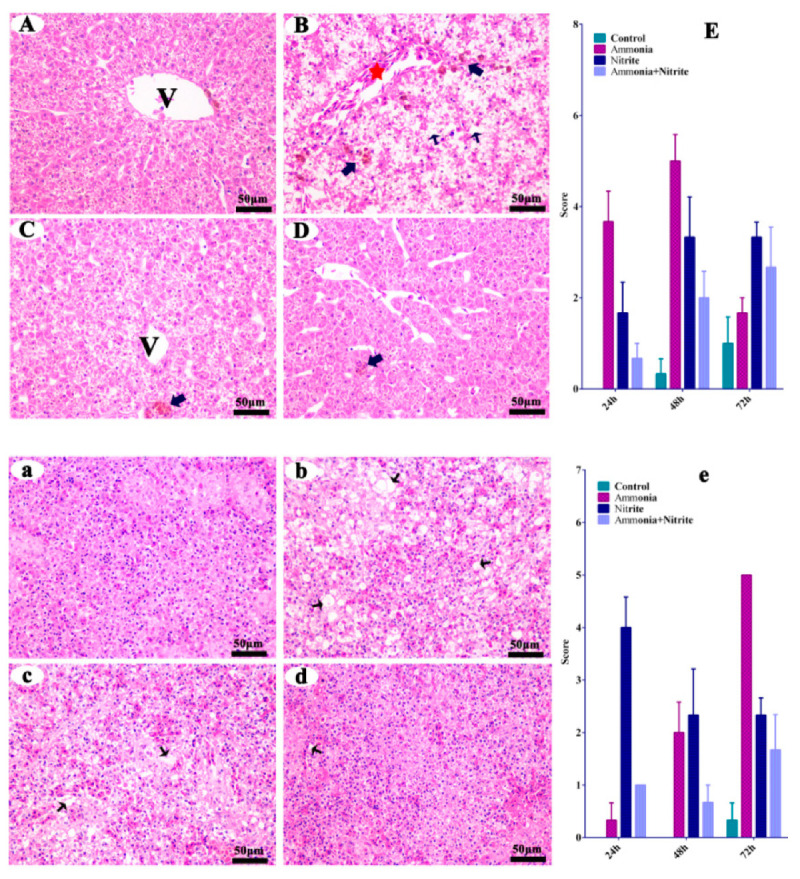
Silver carp hepatic (**A**–**D**) and splenic (**a**–**d**) histological changes in silver carps after ammonia and nitrite exposure. Quantitative analysis of histopathological changes by cytoplasm vacuolization, and congestion of the veins and sinusoids in the liver (**E**) and spleen (**e**) of silver carp during exposure of ammonia, nitrite and the two combined. Error bars show the standard errors (SEs) of the means of three replicates. Thin arrows: cytoplasm vacuolization; bold arrows: melano-macrophage center; red stars: congestion of veins and sinusoids. V: central vein. Scale bar = 50 μm.

The liver and spleen from the control fish did not show any histopathological changes (Figure 1A,a). When the fish were treated with ammonia, hepatic pathological alterations occurred, characterized by cytoplasm vacuolization (CV), melano-macrophage centers (MMC) and the congestion of veins and sinusoids (CVS) (Figure 1B). Similarly, the MMC and CVS were observed in the liver after nitrite stress (Figure 1C). When the fish was treated with a single factor of ammonia or nitrite, splenic pathological alterations were characterized by CV, suggesting that splenic damage was induced by ammonia and nitrite stress (Figure 1b,c). Additionally, less tissue damage was observed in the combined group than in the single-factor groups (Figure 1D,d), indicating the antagonistic effects on hepatic and splenic damage between ammonia and nitrite.

### 3.2. Effects of Ammonia, Nitrite and a Combination of the Two on Antioxidative and Immune Responses

**Figure 2:**
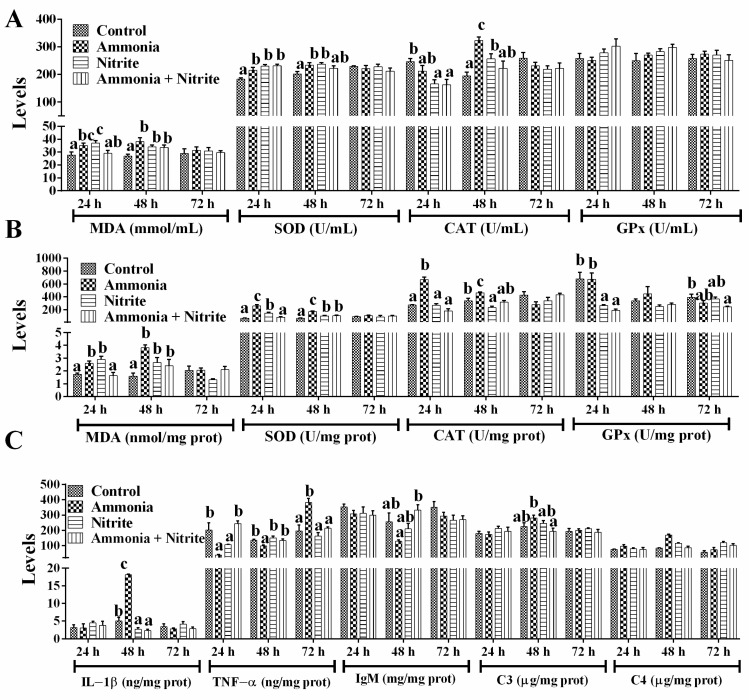
Effects of ammonia, nitrite and their combination on plasmatic parameters (**A**), liver parameters (**B**) and spleen parameters (**C**) after 72 h of exposure. Error bars show the SEs of the means of six replicates in plasma. Different letters above the bars represent significant differences (*p* < 0.05).

In plasma, MDA levels increased significantly in the ammonia and nitrite groups after 24 h and 48 h of exposure, but they only increased significantly at 48 h in the combined group (Figure 2A; *p* < 0.05). After ammonia or nitrite exposure, SOD activities increased significantly at 24 h and 48 h (*p* < 0.05). In the combined group, SOD activities increased significantly at 24 h (*p* < 0.05). The CAT activities decreased significantly in the nitrite and combined groups at 24 h and increased significantly in the ammonia and nitrite groups at 48 h (*p* < 0.05). There was no significant difference in GPx activity between the control and treatment groups (*p* < 0.05). Our results suggested that the SOD-CAT response against ROS toxicity played an important role in the antioxidant response of silver carp under ammonia and nitrite stress conditions. The combination of ammonia and nitrite decreased or recovered SOD and CAT activities induced by ammonia or nitrite exposure. There were statistically significant interactions between ammonia and nitrite for plasmatic MDA and SOD (Table 1; *p* < 0.05). Therefore, both nitrite and ammonia induced oxidative stress and an antioxidant response in silver carp, and they had antagonistic effects.

In the liver (Figure 2B), MDA levels increased significantly in the ammonia and nitrite groups after 24 h and 48 h of exposure; however, in the combined group, MDA only increased at 48 h (*p* < 0.05). Similarly, SOD activities increased significantly in the ammonia and nitrite groups at 24 h and 48 h, and in the combined group only at 48 h (*p* < 0.05). CAT activities increased significantly in the ammonia group at 24 h and 48 h and decreased significantly in the nitrite group at 48 h (*p* < 0.05). A significant decrease in hepatic GPx activity was observed in the nitrite and combined groups (*p* < 0.05). Thus, the SOD-CAT response against ROS toxicity played an important role in the antioxidant response of the liver under ammonia and nitrite stress. Moreover, the combination of ammonia and nitrite decreased or recovered SOD and CAT activities induced by ammonia or nitrite exposure individually. There were significant interactions between ammonia and nitrite for MDA, SOD and CAT (Table 1; *p* < 0.05). Therefore, nitrite and ammonia induced the oxidative stress and antioxidant response in the liver and had an antagonistic effects.

In the spleen (Figure 2C), ammonia significantly increased IL-1β levels, while a decrease in the IL-1β was induced by nitrite and combined exposure after 48 h exposure (*p* < 0.05). Additionally, ammonia induced a significant decrease in TNF-α at 24 h and 48 h, and a significant increase at 72 h (*p* > 0.05). In terms of adaptive immunity, IgM showed no significant differences between the treatment groups and the control group (*p* > 0.05). There were also no significant differences in C3 levels after ammonia and nitrite exposure (*p* > 0.05). However, C4 levels increased significantly after ammonia exposure at 24 h and after nitrite exposure at 24 h and 48 h (*p* < 0.05). Additionally, the combination of ammonia and nitrite mitigated the changes in C3, C4, IL-1β, TNF-α and IgM induced by ammonia or nitrite, indicating antagonistic effects between ammonia and nitrite. Consistent with these results, there were significant interactions of ammonia and nitrite in terms of the splenic immune parameters (Table 1; *p* < 0.05).

### 3.3. Effects of Ammonia, Nitrite and the Combination of the Two on Plasmatic Ion Concentrations and Nitrogen Metabolism

**Figure 3:**
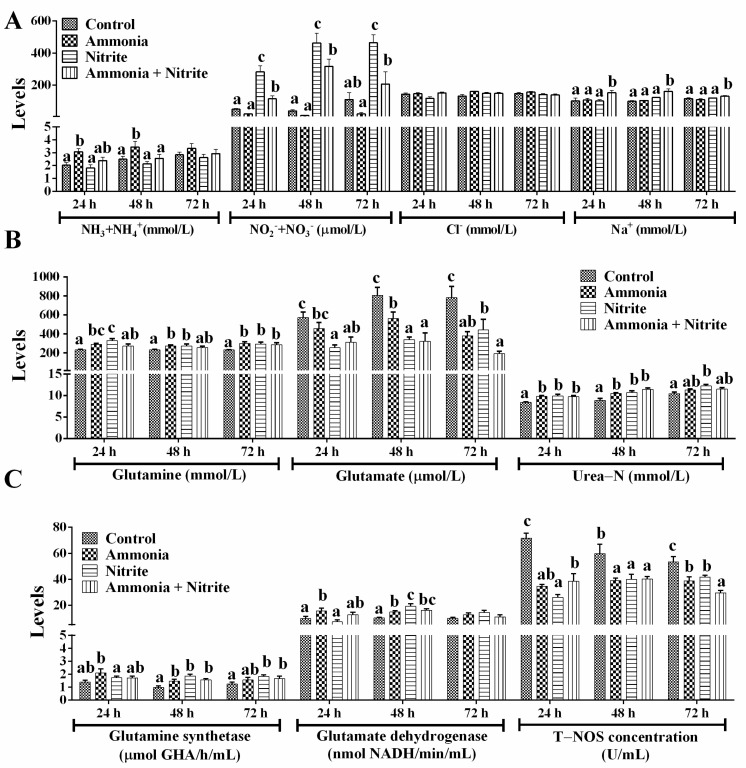
Effects of ammonia, nitrite and a combination of the two on plasmatic ion concentration (**A**) and nitrogen metabolism (**B**,**C**) after 72 h of exposure. Error bars show the SEs of the means of three replicates in the liver and spleen. Different letters above bars represent significant differences (*p* < 0.05).

Plasmatic ammonia (NH_3_+NH_4_^+^) levels increased significantly after ammonia exposure at 24 h and 48 h (Figure 3A; *p* < 0.05) but did not change significantly in the nitrite and combined groups, suggesting that nitrite decreased the plasmatic ammonia increased by ammonia exposure. Plasmatic NO_2_^−^+NO_3_^−^ increased significantly during 72 h of nitrite exposure and during 48 h of ammonia + nitrite exposure (*p* < 0.05) but decreased during 72 h of ammonia exposure (*p* > 0.05). Meanwhile, NO_2_^−^+NO_3_^−^ levels were significantly lower in the combined group than in the nitrite group (*p* < 0.05), indicating that ammonia decreased the level of plasma NO_2_^−^+NO_3_^−^ increased by nitrite. This phenomenon indicated that nitrite and ammonia might alleviate each other’s toxicity. Consistent with these results, a significant interaction between ammonia and nitrite on plasma NO_2_^−^+NO_3_^−^ was observed (Table 1). After 72 h of exposure, plasmatic Na^+^ was not significantly different between ammonia/nitrite groups and the control group (*p* > 0.05) but was increased significantly in combined group (*p* < 0.05). There was a significant interaction between ammonia and nitrite for plasma Na^+^ at 72 h (Table 1). However, there were no significant changes in plasma Cl^−^ between the treatment groups and the control group (*p* > 0.05).

After ammonia and nitrite exposure, Gln and Glu levels, respectively, increased and decreased significantly (Figure 3B; *p* < 0.05). In the combined group, Glu levels decreased significantly during 72 h of exposure, but Gln levels increased significantly at 72 h (*p* < 0.05). Plasmatic urea-N levels increased significantly in the three treatment groups compared with those in the control group at 24 h and 48 h (*p* < 0.05). The activities of GS and GDH increased significantly in the ammonia group at 24 h and 48 h (Figure 3C; *p* < 0.05). In the nitrite and combined groups, GS activities increased significantly at 48 h and 72 h, and GDH activities increased significantly at 48 h (*p* < 0.05). Overall, internal ammonia was detoxified directly into Gln and urea in silver carp to cope with ammonia and nitrite exposure. Additionally, T-NOS activities decreased significantly in the three treatment groups compared with those in the control group after 72 h of exposure (*p* < 0.05), indicating that nitrite and ammonia might reduce the accumulation of NO_2_^−^+NO_3_^−^ by decreasing their biosynthesis via NO. Two-way ANOVA analysis indicated that ammonia and nitrite had significant interactions for urea-N, T-NOS, Gln, GDH and GS (Table 1; *p* < 0.05). Compared with single-factor ammonia or nitrite, their combined toxicities toward T-NOS, Glu, GDH, Gln, urea-N and GS were lower. Therefore, the antagonistic toxicity of ammonia and nitrite in *H. molitrix* could involve decreases in Gln and NO synthesis and metabolism under their combined exposure.

### 3.4. Transcriptional Characteristics

Clean reads Q20 (%) and clean reads Q30 (%) values of all samples from the liver and spleen exceeded 97.94% and 94.02%, respectively, showing that the majority of raw reads generated by high-throughput sequencing passed quality filtering (Table S2). After quality filtering, paired clean reads were extracted and mapped to the silver carp genome (unpublished). There were significant separations between the control group and groups with ammonia, nitrite and the two combined in the liver and spleen (Figure S1). Meanwhile, exposure to ammonia, nitrite and their binary combination resulted in a number of DEGs in the liver and spleen, among which the number of downregulated DEGs was much greater than that of upregulated DEGs after 48 h of exposure (Figure S1). In total, 1191, 1772 and 1369 DEGs were identified in the liver after exposure to ammonia, nitrite and the two combined, respectively. In the spleen, we observed 2885, 2686 and 3762 DEGs in the groups with ammonia, nitrite, and the two combined, respectively. Total and overlapping numbers of DEGs among three comparisons are shown in Figure S1.

### 3.5. Functional Annotation and Classification of the Transcriptome

**Figure 4:**
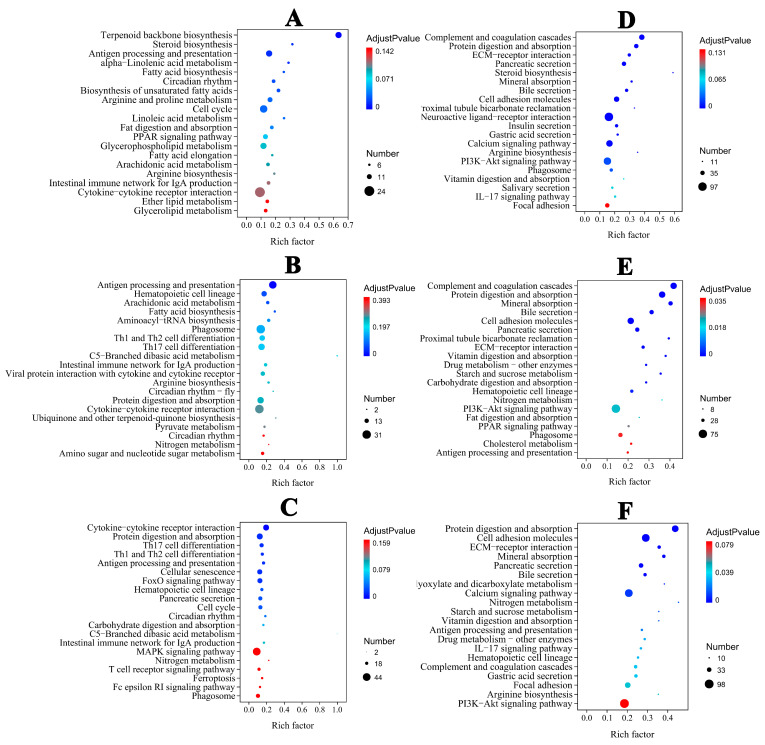
Top 20 KEGG−enriched important pathways. Liver: (**A**) (ammonia), (**B**) (nitrite), (**C**) (ammonia + nitrite). Spleen: (**D**) (ammonia), (**E**) (nitrite), (**F**) (ammonia + nitrite).

In the liver, the majority of DEGs were categorized into biological process (BP) categories, including steroid and lipid metabolism in the ammonia group (Figure S1A). In the nitrite group, the majority of DEGs were categorized into BP categories, including metabolic process, stress and the immune response. In the combined groups, DEGs were categorized into cellular component (CC) and BP categories (Figure S1C). Extracellular region and space were the dominant annotated DEGs in the CC category. In the BP category, the dominant annotated DEGs were associated with immune cell differentiation, activation and regulation. Therefore, BP was significantly categorized in the three treatment groups (Figure S1A–C). In the spleen, the majority of DEGs were categorized into CC, BP and MF categories in the three treatment groups (Figure S1D–F). According to KEGG pathway annotations, in the ammonia and nitrite groups, the DEGs were involved in metabolism and organismal systems, mainly including lipid metabolism, acid amino metabolism, nitrogen metabolism, the proliferator-activated receptor (PPAR) signaling pathway and antigen processing and presentation in the liver and spleen (Figure 4A,B,D,E). However, the maximum number of DEGs were categorized into organismal systems and environmental information processes, including lymphocyte differentiation, protein digestion and absorption, antigen processing and presentation and cytokine–cytokine receptor interactions, in the combined group (Figure 4C,F). These results indicated that there was interaction between ammonia and nitrite.

Herein, we mainly focused on genes related genes to amino acid metabolism and the immune response. In the liver, genes related to amino acid catabolism displayed significantly decreased expression after ammonia or nitrite exposure (Table S3). Therefore, silver carp decreased ammonia production in vivo by down-regulating amino acid metabolism after ammonia and nitrite exposure. However, GS gene expression increased significantly after ammonia and nitrite stress, which indicated that silver carp detoxified ammonia to glutamine when exposed to ammonia and nitrite. Additionally, the expression of genes involved in aspartate and cysteine anabolism increased significantly after nitrite exposure. In addition, combined exposure exacerbated the changes in GS and NOS gene expression induced by ammonia or nitrite (Table S3). In the spleen, genes related to arginine, alanine and glycine catabolism showed a significant decrease, whereas those related to catabolism of glutamine and methionine showed a significantly increased expression after ammonia and nitrite exposure (Table S3). Additionally, more amino acid catabolism-associated genes were decreased by combined exposure.

For the DEGs related to the immune response, the downregulated expression of the genes encoding tumor necrosis factor superfamily member 2, transcription factor AP-1, protein Fos B, transcription factor jun-D in liver, TNF-α, interleukin 6 signal transducer, toll-like receptor 4, IL-4R, proto-oncogene protein c-fos and P38 MAP kinase in spleen were observed (Table S3). However, the expression levels of genes encoding complement component genes (C3, C4, C8g, C9) in the spleen of silver carp increased significantly after single-factor ammonia or nitrite exposure. Additionally, there was an antagonistic effect on the DEGs related to the immune response between ammonia and nitrite (Table S3).

### 3.6. Verification DEGs Using qRT-PCR

**Figure 5:**
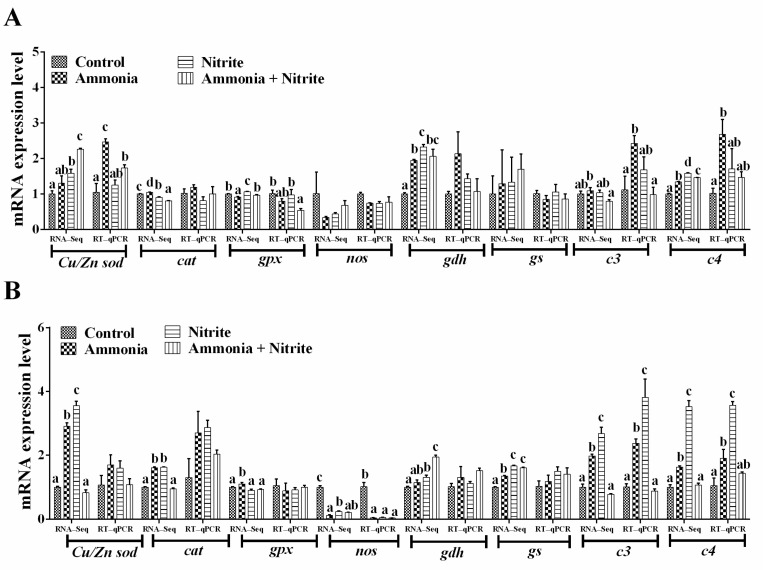
Quantitative real-time reverse transcription PCR verification of the transcriptome data in the liver (**A**) and spleen (**B**). Different letters above bars represent significant differences (*p* < 0.05).

Eight genes involved in the antioxidative response (*Cu/Znsod*, *cat*, *gpx*), nitrogen metabolism (*gdh*, *gs*, *nos*) and the immune response (*c3*, *c4*) were used for qRT-PCR verification (Figure 5A,B). We observed the same expression trends of these genes between the qRT-PCR and transcriptome analysis results. Additionally, their expression trend was consistent with their protein level changes in the liver, spleen or plasma (Figure 2 and Figure 3C). Significant increases in hepatic *Cu/Zn sod*, *c3* and *c4* were observed in silver carp after ammonia exposure (*p* < 0.05). However, the levels of hepatic *gpx* decreased significantly after co-exposure to ammonia and nitrite (*p* < 0.05). In the spleen, nos expression decreased significantly in the three treatment groups compared with the control group (*p* < 0.05). Meanwhile, significant increases in c3 and *c4* expression levels were observed after ammonia or nitrite exposure (*p* < 0.05).

### 3.7. IBR Indices

**Figure 6:**
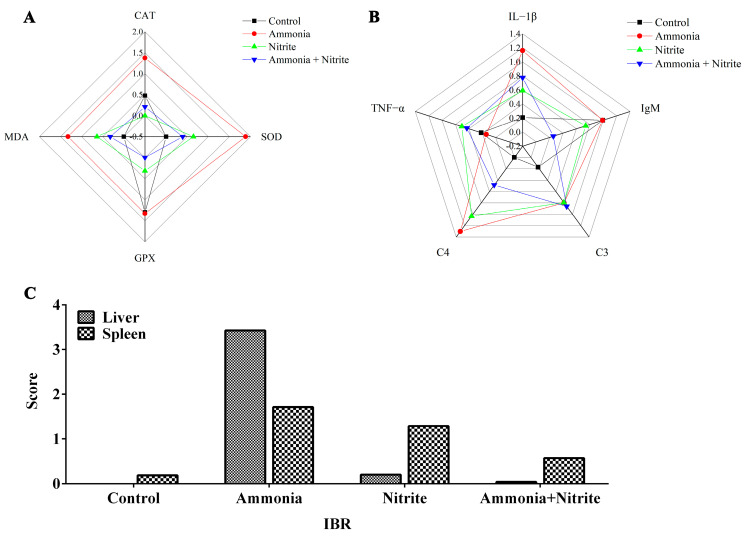
Integrated biomarker response analysis (IBR) analysis in silver carps after exposure to ammonia, nitrite and a combination of the two. Star plots: (**A**) liver; (**B**) spleen. IBR values (**C**).

The IBR values were calculated from the standardized data of four and five biomarkers in the liver and spleen, respectively (Figure 6). The IBR values were increased by ammonia and nitrite exposure in the liver and spleen, indicating that in fish, those organs are the potential targets for ammonia and nitrite toxicity. Additionally, the IBR values in the liver and spleen were higher in the ammonia group than in the nitrite group, suggesting that ammonia might be more toxic than nitrite to the liver and spleen in silver carp. Moreover, IBR values were lower after co-exposure than after exposure to ammonia and nitrite individually, indicating the antagonistic effects of nitrite and ammonia.

## 4. Discussion

Silver carp exposed to ammonia or nitrite displayed significant pathological changes to cytoplasmic vacuolization, the congestion of veins and sinusoids and the melano-macrophage center in the liver. Similarly, changes to cytoplasmic vacuolization and veins and sinusoids in the liver were documented in fish exposed into either ammonia [32] or nitrite [36]. Cytoplasmic vacuolization might be associated with oxidative stress under nitrite and ammonia exposure [37]. Herein, plasmatic MDA levels increased significantly after ammonia and nitrite exposure in our present study, suggesting that oxidative stress was responsible for the observed cytoplasmic vacuolization. The melano-macrophage center comprises macrophage aggregates, whose increased size and frequency indicate that fish are experiencing oxidative stress or damage [8]. Increased melano-macrophage center was reported in the spleen of *Megalobrama amblycephala* after ammonia exposure [38]. In the spleen, cytoplasmic vacuolization was induced by ammonia and nitrite in the present study. Similar results were observed in largemouth bass and pigs exposed to nitrite and ammonia, respectively [39,40]. Therefore, cytoplasmic vacuolization was possibly the common mode of action of the oxidative stress induced by ammonia and nitrite in the liver and spleen. 

Additionally, ammonia toxicity was more severe in the liver than in the spleen; however, the opposite result was induced by nitrite based on pathological changes and IBR values. Similarly, damage to the liver induced by ammonia was more severe than that in the muscle, gill and kidney of *Dicentrarchus labrax* [41]. Ammonia mainly causes oxidative stress in organisms by increasing ROS levels, whose production rate in a metabolically dynamic tissue (liver) appeared to be higher compared with that in average oxygen consuming tissue (spleen, gill and muscle) [41]. The spleen, which plays a vital role in blood filtration and the immune response, easily increases its filtration burden by elevating the blood methemoglobin level in response to nitrite stress. In this study, there was an antagonistic effect of ammonia and nitrite on the pathological changes and IBR values. Brazão et al. also reported that the combination of ammonia and nitrite induced antagonistic effects on gill damage in *M. amazonicum* [22]. However, in *L. vannamei*, their combined stress caused more extensive histopathological damage to the hepatopancreas compared with either stress individually [20,42]. Thus, our results showed that single ammonia or nitrite exposure caused pathological damage through oxidative stress; however, they had an antagonistic effect.

In the liver, MDA (a lipid peroxidation product) levels increased significantly in silver carp exposed to either ammonia and nitrite alone, indicating that ammonia and nitrite induced oxidative stress. Similarly, increased hepatic MDA levels were reported in sea bass and *Siniperca chuatsi* under ammonia and nitrite exposure, respectively [41,43]. In our study, significant increases in SOD and CAT activities in the liver and plasma were induced by ammonia alone. In response to ROS production, SOD catalyzes the conversion of O_2_-· to H_2_O and H_2_O_2_, and the latter is further degraded into H_2_O and O_2_ by CAT or GPx. Thus, SOD and CAT provide the first line of defense against oxygen toxicity. Our results suggested that only SOD-CAT defense against ROS toxicity was activated by ammonia in silver carp. Similarly, ammonia only initiated SOD-CAT defense in *M. amblycephala* [32]. By contrast, treatment with nitrite alone caused significant increase in SOD activity but decreases in CAT and GPx activities in plasma or the liver, indicating that SOD might play a vital role in the antioxidative response to nitrite toxicity in silver carp. The CAT and GPx activities decreased significantly in *S. chuatsi* exposed to nitrite [43]. However, the combination of nitrite and ammonia decreased/recovered the increases in MDA levels and SOD and CAT activities induced by ammonia or nitrite alone. Moreover, significant interactions were determined between ammonia and nitrite for plasmatic MDA and SOD and for hepatic MDA, SDO and CAT. Thus, ammonia-initiated SOD-CAT defense and nitrite individually activated the SOD antioxidant response in silver carp, and they had antagonistic effects on the oxidative and antioxidative responses. In contrast, ammonia and nitrite had synergistic effects on the oxidative and antioxidative response in *L. vannamei* [19]. 

Our transcriptomic analysis indicated that hepatic DEGs induced by ammonia were mainly involved in energy production, the amino acid metabolic rate and the immune response to hepatic toxicity. The large numbers of DEGs induced by acute ammonia administration were involved in amino acid metabolism pathways in the liver of *Largescale loach* [44] and in the gills of *Solenaia oleivora* [45]. Herein, after nitrite exposure, hepatic DEGs were mostly enriched in pathways of metabolism and immune defense. A similar response was reported in *Litopenaeus vannamei* and *M. amblycephala* under nitrite stress [37]. Taken together, oxidative stress and the disruption of metabolism and the immune response were the common features of ammonia and nitrite hepatotoxicity; however, they had antagonistic effects on hepatotoxicity.

In the spleen, the immunity- and metabolism-related pathways were mainly enriched by DEGs under stress of ammonia and nitrite individually. Similar results were observed in the hepatopancreas of *Scylla paramamosain* under ammonia exposure [46] and in *M. nipponense* after nitrite stress [47]. The complement and coagulation cascades pathways were enriched with the most immunity-related DEGs in silver carp under exposure to ammonia, nitrite and a combination of the two. Splenic C4 levels and the transcription levels of *c4*, *c3*, *c8g* and *c9* increased significantly after exposure to ammonia and nitrite alone. Similarly, ammonia exposure up-regulated hepatic C3 levels in *Carassius gibelio* [48]. IL-1β and TNF-α are the first cytokines released during inflammation in fish. In the present study, splenic IL-1β levels were increased and decreased by ammonia and nitrite, respectively. Similarly, ammonia induced the release of IL-1β in zebrafish and increased *il-1β*, *il-6* and *il-12* transcription in *Takifugu obscurus* [7,49]; however, nitrite significantly decreased the transcription of splenic *il-1β* and *tnf-α* in zebrafish [36]. Additionally, ammonia induced a significant decrease in splenic TNF-α during 48 h of exposure, but it was increased at 72 h in the present study. Similarly, the expression levels of *tnf-α* initially showed an increasing trend and then decreased in *Poecilia reticulate* after ammonia stress [50]. However, splenic TNF-α levels only decreased significantly after 24 h of nitrite stress, which was consistent with the significant decrease in *tnf-α* expression in the gills of *Scophthalmus maximus* after nitrite exposure [51]. Guo et al. also reported that ammonia caused marked decreases in splenic IL-1β and TNF-α levels in Wuchang bream [8]. Additionally, the combination of ammonia and nitrite mitigated the changes induced by ammonia or nitrite individually. Therefore, the disruption of immune responses and metabolism plays an important role in the toxicity of nitrite and ammonia in the spleen, and nitrite and ammonia had an antagonistic effect.

Although the combined toxicity of nitrite and ammonia on shrimp has been a concern in the past, the toxicological mechanism of their combination is unclear. In the present study, ammonia exposure increased plasmatic NH_3_+NH_4_^+^ but decreased plasmatic NO_2_^−^+NO_3_^−^. Similarly, increased ammonia concentrations were detected in the spleen and head kidneys of *M. amblycephala* exposed to ammonia [8]. However, in the present study, nitrite exposure decreased plasmatic NH_3_+NH_4_^+^ levels and increased plasmatic NO_2_^−^+NO_3_^−^ levels. Therefore, the competitive accumulation of ammonia and nitrite likely caused their antagonistic effects. Higher tolerance to nitrite in fish might contribute to the competitive effect between NO_2_^−^ and Cl^−^. However, there were no changes in plasmatic Cl^−^ after exposure to a combination of ammonia and nitrite in the present study. Similarly, plasmatic Na^+^ levels were not significantly altered after nitrite or ammonia exposure, which is consistent with previous studies on turbot after ammonia exposure [52] and on *Sparus sarba* after nitrite exposure [53]. However, plasmatic Na^+^ levels increased significantly after co-exposure to ammonia and nitrite in the present study. This result indicated that the competitive effect between NH_4_^+^ and Na^+^ on either exchanger in gill might be affected by combined exposure because NH_4_^+^ is excreted through the gills mainly via a carrier-mediated process in exchange for Na^+^. However, future studies should clarify the mechanism by which plasmatic Na^+^ levels increased significantly after co-exposure to ammonia and nitrite.

**Figure 7:**
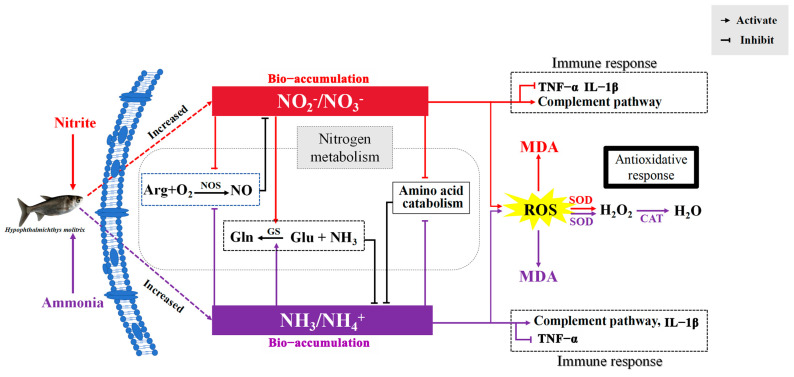
Schematic summary of the proposed mechanism for the antagonistic effects of ammonia and nitrite in silver carp. ARG, arginine; NO, nitric oxide; NOS, nitric oxide synthase; Gln, glutamine; Glu, glutamate; GS, glutamine synthetase; ROS, reactive oxygen species; MDA, malondialdehyde; SOD, superoxide dismutase; CAT, catalase; TNF-α, tumor necrosis factor α; IL-1β, interleukin 1β. Red lines indicate ammonia nitrogen effects, purple lines indicate nitrite effects and black lines indicate the effects of the two combined.

Plasmatic glutamine and urea-N increased significantly after nitrite and ammonia exposure, indicating that ammonia could be detoxified directly into glutamine and urea in silver carp to cope with ammonia and nitrite stress. Fish can usually detoxify ammonia to glutamine and urea under ammonia loading [54]. Generally, the detoxification of ammonia via glutamine relies largely on the activity of glutamine synthetase (GS). In the present study, the activities of GS and GDH increased as the level of glutamate in plasma decreased following nitrite or ammonia exposure. Additionally, the significantly increased transcription of the gene encoding GS was induced by ammonia or nitrite in the spleen and liver. Therefore, nitrite was able to decrease the accumulation of ammonia by increasing the biosynthesis of glutamine and urea. Similarly, nitrite enhanced ammonia-induced increases in nitrogen metabolism and the production of urea-N and other organic-N in *M. japonicus* [18]. The existence of another mechanism for detoxifying ammonia by decreasing endogenous ammonia production was also proven in fish [55]. In the present study, amino acid metabolism decreased significantly under ammonia and nitrite exposure, indicating that a decrease in endogenous ammonia production was another strategy to detoxify ammonia in silver carp under ammonia and nitrite stress. Additionally, plasmatic T-NOS activity decreased significantly after ammonia and nitrite exposure, indicating that ammonia was able to decrease the accumulation of NO_2_^−^+NO_3_^−^ via nitric oxide (NO). In vivo, NO_3_^−^ and NO_2_^−^ are formed by the oxidation of NO, which is produced from the conversion of L-arginine to NO and citrulline in the presence of NOS in the arginine pathway. A significant decrease in the NO content in the brain of *Micropterus salmoides* was induced by 24 h of ammonia exposure [56], and hepatic NOS activity decreased significantly under ammonia stress in *P. fulvidraco* [57]. In addition, decreased transcription of *nos* was also induced by ammonia and nitrite in the spleen and liver, respectively. Taken together, the antagonistic effects on silver carp induced by ammonia and nitrite can be explained by their competitive bio-accumulation of ammonia and nitrite in *H. molitrix* (Figure 7). Therefore, the simultaneous monitoring and control of both ammonia and nitrite concentrations are essential to mitigate their compounded toxic effects, which might be exacerbated under isolated exposure conditions.

## 5. Conclusions

In the present study, exposure to ammonia or nitrite individually caused pathological damage through oxidative stress. Cytoplasmic vacuolization was the common effect of ammonia and nitrite in the liver and spleen. Ammonia toxicity was more severe in the liver than in the spleen; however, nitrite induced the opposite results. Ammonia initiated the SOD-CAT defense, whereas nitrite only activated the SOD antioxidant response in silver carp. In the liver, oxidative stress and disrupted metabolisms and immune responses were the common features of ammonia and nitrite hepatotoxicity; however, they had antagonistic effects on hepatotoxicity. In the spleen, the disruption of the immune responses and metabolism played an important role in the toxicity of nitrite and ammonia, where they also had antagonistic effects. Moreover, the antagonistic effect of nitrite and ammonia on liver and spleen could be explained by their competitive bio-accumulation in silver carp (Figure 7). Therefore, ammonia and nitrite should be managed simultaneously to avoid the more intense toxicity induced by their combination. However, to obtain more detailed information regarding the interaction effects of nitrite and ammonia on fish physiology, we suggest that future research should measure the chronic toxicities of ammonia and nitrite on fish for comparative analysis to validate the present results.

## Figures and Tables

**Table 1:** Two-way analysis of variance of the interaction between nitrite and ammonia in terms of liver, spleen and plasma parameters of silver carp after 72 h of exposure.

Tissue	Parameter	Time					
		24 h		48 h		72 h	
		F	*p*	F	*p*	F	*p*
Plasma	MDA	12.289	0.002	7.694	0.012	0.447	0.511
	SOD	5.246	0.033	6.952	0.016	0.194	0.664
	CAT	0.523	0.478	1.922	0.181	0.317	0.580
	GPx	0.694	0.415	0.023	0.882	1.269	0.273
	Ammonia	0.803	0.381	0.327	0.574	0.026	0.872
	NO_2_^−^+NO_3_^−^	10.169	0.005	2.317	0.144	2.761	0.112
	Cl^−^	0.357	0.557	2.459	0.133	4.305	0.051
	Na^+^	3.16	0.091	4.079	0.057	6.305	0.020
	Urea-N	6.968	0.016	5.314	0.032	1.464	0.240
	T-NOS	44.290	0.000	5.882	0.025	0.242	0.628
	Glutamate	3.243	0.088	1.466	0.241	0.707	0.411
	Glutamine	12.724	0.002	5.005	0.037	4.399	0.049
	GDH	0.011	0.919	8.461	0.009	4.400	0.049
	GS	3.132	0.092	8.498	0.009	2.192	0.154
Liver	MDA	27.632	0.001	2.813	0.132	0.120	0.738
	SOD	88.546	0.000	28.956	0.001	0.001	0.972
	CAT	66.294	0.000	6.459	0.035	0.459	0.517
	GPx	0.227	0.646	0.415	0.537	0.183	0.680
Spleen	IL-1β	0.070	0.797	138.794	0.000	0.327	0.583
	TNF-α	34.671	0.000	0.956	0.357	6.575	0.033
	IgM	0.344	0.574	10.303	0.012	0.974	0.352
	C3	0.093	0.769	6.998	0.033	1.337	0.285
	C4	2.473	0.154	62.041	0.000	2.231	0.174

Note: MDA, malondialdehyde; SOD, superoxide dismutase; CAT, catalase; GPx, glutathione peroxidase; T-NOS, total nitric oxide synthase; GDH, glutamate dehydrogenase; GS, glutamine synthetase; IL-1β, interleukin 1 beta; TNF-α, tumor necrosis factor alpha; IgM, immunoglobulin M.

## Data Availability

The raw data supporting the conclusions of this article will be made available by the authors without undue reservation.

## Supplementary Materials

The following supporting information can be downloaded at: https://www.mdpi.com/article/10.3390/antiox14040453/s1. Text S1. Histopathological quantification evaluation; Text S2. Total RNA extraction and transcriptome sequencing; Text S3. Integrated biomarker response analysis; Table S1. Sequences of primers used for real-time PCR in head kidneys; Table S2. Summary of the sequencing data quality and clean reads mapped to the reference genome; Table S3. List of the DEGs involved in amino acid metabolism and immune response between control and treatment groups according to KEGG pathway annotations. Figure S1. Transcriptomic analyses in silver carps after exposure to ammonia, nitrite and mixture of the two for 48 h. (A: liver, F: spleen) Principal component analysis (PCA); (B: liver; G: spleen) different numbers of DEGs; (C: liver; H: spleen) Venn diagram of DEGs; (D: liver; E: spleen) hierarchical clustering for the differentially expressed genes. Figure S2. Go enrichment analysis bar chart (Liver: A–C; Spleen: D–F) CC: cellular component; BP: biology process; MF: molecular function.
