# Supplementary material for: Competitive Bio-Accumulation Between Ammonia and Nitrite Results in Their Antagonistic Toxicity to Hypophthalmichthys molitrix: Antioxidant and Immune Responses and Metabolic Detoxification Evidence
Source: Antioxidants (Basel). 2025 Apr 10;14(4):453. doi: 10.3390/antiox14040453 (PMC12024166; doi:10.3390/antiox14040453)
Supplement: Supplementary file 1 [file antioxidants-14-00453-s001.zip › antioxidants-3551583-supplementary.pdf]

Competitive Bio-accumulation Between Ammonia and Nitrite Causes Their Antagonistic Toxicity to *Hypophthalmichthys molitrix*: Antioxidant and Immune Response and Metabolic detoxification Evidence

Honghui Guo[1], Yiwen Li[1,2], Heng Ge[1], Hang Sha[1], Xiangzhong Luo[1], Guiwei Zou[1], Hongwei Liang[1,2*]

[1] Yangtze River Fisheries Research Institute, Chinese Academy of Fisheries, Wuhan,

[2] China College of Fisheries, Huazhong Agricultural University, Wuhan 430070, China;

***Correspondence:**

Dr. Hongwei Liang,

Email: lianghw@yfi.ac.cn

Text S1. Histopathological quantification evaluation

Liver and spleen samples were cut as size as 3-5 mm^3 and fixed in 10% neutral-buffered formalin. After 48-hour fixation, they were processed routinely including embedded in paraffin wax, sectioned (5 μm), and stained with haematoxylin and eosin (H&E). Histopathological assessment was done on a light microscopy (Nikon H600L Microscope and image analysis system, Tokyo, Japan). Histological changes were further quantitatively evaluated according to a protocol proposed by Bernet et al., (1999) and Corbett et al., (2015). Images of liver and spleen section were captured at 400 x magnification. Three images per tissue section were randomly selected for quantification analysis. Histological alterationwas quantified using either the numerical occurrence of a particular alterationper unit area, or the percentage cover of alteration depending on the alteration and organ. A severity score value from 0 to 6 was assigned for the degree and extent of each alteration: 0 for unchanged tissue, (1 or 2) for mildly increased cytoplasm vacuolization, melano-macrophage centre, and congestion of vein and sinusoid, (3 or 4) for moderately increased cytoplasm vacuolization, melano-macrophage centre, and congestion of vein and sinusoid, (5 or 6) for severely increased cytoplasm vacuolization, melano-macrophage centre, and congestion of vein and sinusoid. Severity score values are based on numeric quantification (either % cover or number per unit area).

Text S2 Total RNA extraction and transcriptome sequencing

TRIzol reagent (Invitrogen, Waltham, MA, USA) was used to extract total RNA from samples of liver and spleen according to the supplier's guidelines. Bioanalyzer 2100 (Agilent Technologies, Santa Clara, CA, USA) and 1% agarose gel electrophoresis were used to assess the integrity and quality of the RNA, respectively. NanoDrop instrument (Thermo Scientific, Waltham, MA, USA) was then used to precisely determine the

RNA concentration. RNA sequencing (RNA-seq) libraries were prepared using 122-μg RNA samples from the control and treatment groups, employing a TruSeq™ RNA Sample Preparation Kit (Illumina, San Diego, CA, USA). Library sequencing was accomplished using paired-end sequencing on the Illumina HiSeq 2500 sequencing platform. Sequencing data can be accessed at the NCBI SRA database (CRA015776). Shanghai Paisennuo Biotechnology Co. Ltd. (Shanghai, China) analyzed the RNA-Seq data bioinformatically. Identified differentially expressed genes (DEG) were subjected to gene ontology (GO) analysis and Kyoto Encyclopedia of Genes and Genomes (KEGG) enrichment analysis in the KOBAS software (version 2.1.1) with statistical significance being indicated by a P value < 0.05. KEGG enrichment analysis results were then used for pathway integration analysis.

Text S3. Integrated biomarker response analysis

The procedure of IBR calculation was briefly described here: (1) Data were standardized by the formula $Y = (X - m)/s$, where X is the value of each biomarker response, m is the mean value of the biomarker, and s is the standard deviation of the biomarker. (2) Using standardized data, Z was calculated as $Z = Y$ in the case of activation or $Z = -Y$ in the case of inhibition. Thus, the minimum value (Min) was obtained for each biomarker. (3) The score (S) was computed as $S = Z + |Min|$, where $S \geq 0$ and $|Min|$ is the absolute value of Min. (4) Calculation of star plot areas by multiplying the obtained value of each biomarker (Si) with the value of the next biomarker, arranged as a set, dividing each calculation by 2. (5) Summing up all values, and the corresponding IBR value is obtained as $IBR = \{[(S1 \times S2)/2] + [(S2 \times S3)/2] +\ldots [(Sn\text{-}1 \times Sn)/2]\}$. IBR/n = IBR/ the number of test parameters.

Table S1. Sequences of primers used for Real-time PCR in head kidney

Target	Forward primer	Annealing T_m
sod	F: TCTCGGGTGAAATCACTGGC	55
	R: TGATGCAGCCGTTTGTGTTG	
cat	F: GCGGAGAACTGGAAGTGGAA	55
	R: GCCGATGTGTGTCTGGGTAA	
gpx	F: TCACTGTGCTTGGATTCCCC	55
	R: CCGTTCACCTCAATCCTGCT	
nos	F: GCATGGAGAAATGCAGCTCG	57
	R: ATCTCGGTCCCCATGTACCA	
gdh	F: GGCTGGCCTTACCTTCACAT	57
	R: AAACGTGAGGTGCTGTAGGG	
gs	F: AGTCGTGTGTGAAGCCTCTG	57
	R: CCACGTGAAAGGCAAACTGG	
c3	F: GATTGCCATGCAAGAGAGCG	55
	R: CTTCTCAACAGGTCTGGCGT	
c4	F: TCTCGCACTCAACCACAGTC	55
	R: GTTTGTTGCGCCTAAAGCCA	
β-actin	F: TGTGACGACCCAAGTCTCCCTT	56
	R: CTGTGGCTCTCCTCCACCATTC	

Table S2. Summary of the sequencing data quality and clean reads mapped to the reference genome

Tissue	Sample	Raw read number	Clean reads	Raw Q20 rate	Raw Q30 rate	Total mapped	Mapped to gene	Mapped to interGene	Mapped to exon
Liver	Control-1	43916100	43537368	98.3	94.8	41185628 (94.60%)	36500427 (93.98%)	2338477 (6.02%)	35825995 (98.15%)
	Control-2	43242986	42899784	98.44	95.21	40622607 (94.69%)	36020038 (93.91%)	2334272 (6.09%)	35321825 (98.06%)
	Control-3	50322604	49753642	98	94.14	47118138 (94.70%)	41643076 (93.83%)	2736912 (6.17%)	40852450 (98.10%)
	Ammonia-1	45595900	45242820	98.57	95.43	42243337 (93.37%)	37170811 (93.72%)	2490773 (6.28%)	36547897 (98.32%)
	Ammonia-2	43046000	42766194	98.68	95.63	40116019 (93.80%)	35254956 (93.74%)	2354719 (6.26%)	34687116 (98.39%)
	Ammonia-3	48447416	48128892	98.71	95.8	45189896 (93.89%)	39663740 (93.82%)	2614092 (6.18%)	39056426 (98.47%)
	Nitrite-1	44425894	44036170	98.36	95.02	41161445 (93.47%)	36200108 (93.50%)	2514679 (6.50%)	35498919 (98.06%)
	Nitrite-2	44580226	44205708	98.25	94.67	41344093 (93.53%)	36450462 (93.66%)	2467261 (6.34%)	35786733 (98.18%)
	Nitrite-3	46715772	46277500	98.21	94.6	43310475 (93.59%)	38098876 (93.55%)	2628451 (6.45%)	37419545 (98.22%)
	Ammonia+Nitrite-1	49360254	48976714	98.38	95.02	45653830 (93.22%)	40294985 (93.99%)	2577988 (6.01%)	39622516 (98.33%)
	Ammonia+Nitrite-2	43081984	42656484	98.18	94.53	39801070 (93.31%)	35063938 (93.87%)	2289370 (6.13%)	34452454 (98.26%)
	Ammonia+Nitrite-3	48728710	48195230	98.01	94.12	44997823 (93.37%)	39629589 (93.85%)	2594909 (6.15%)	38927474 (98.23%)
Spleen	Control-1	47286956	46942674	98.53	95.32	43951477 (93.63%)	38405650 (91.02%)	3787500 (8.98%)	36613777 (95.33%)
	Control-2	49437838	48997866	98.37	94.89	45868865 (93.61%)	40133042 (91.05%)	3943483 (8.95%)	38138591 (95.03%)
	Control-3	44245534	43870502	98.32	94.72	41046197 (93.56%)	35783061 (90.72%)	3659829 (9.28%)	33820588 (94.52%)
	Ammonia-1	50948528	50403550	98.02	94.11	47752620 (94.74%)	42275339 (92.33%)	3513196 (7.67%)	40721174 (96.32%)
	Ammonia-2	52389432	51901670	98.3	94.91	49014312 (94.44%)	43190395 (91.93%)	3792915 (8.07%)	41403634 (95.86%)
	Ammonia-3	46732962	46163066	97.94	94.02	43944427 (95.19%)	38937175 (92.43%)	3187050 (7.57%)	37661825 (96.72%)
	Nitrite-1	50699836	50177542	98.16	94.54	47203041 (94.07%)	41420971 (91.55%)	3824812 (8.45%)	39571763 (95.54%)
	Nitrite-2	52043486	51534508	98.21	94.62	48698316 (94.50%)	42751051 (91.61%)	3915571 (8.39%)	40951220 (95.79%)
	Nitrite-3	49970642	49510840	98.31	94.93	46817760 (94.56%)	41318519 (92.05%)	3567625 (7.95%)	39594998 (95.83%)
	Ammonia+Nitrite-1	49153614	48689776	98.33	95.05	45798457 (94.06%)	40371965 (91.17%)	3908650 (8.83%)	38119299 (94.42%)
	Ammonia+Nitrite-2	50607568	50141624	98.23	94.67	47261460 (94.26%)	41789058 (91.47%)	3895931 (8.53%)	39650718 (94.88%)
	Ammonia+Nitrite-3	51065456	50527458	98.13	94.51	47608690 (94.22%)	42040108 (91.30%)	4003778 (8.70%)	39756838 (94.57%)

Table S3. List of the DEGs involved in amino acid metabolism and immune response between control and treatment groups according to the KEGG pathway annotations

Tissue	pathway	Genes	Groups					
			Ammonia		Nitrite		Ammonia+Nitrite	
			log2 Fold Change	*P* adj	log2 Fold Change	*P* adj	log2 Fold Change	*P* adj
Liver	Amino acid metabolism	Glutaminase	-2.816	0.006	-		-	
		Glutamine synthetase	1.397	0.000	1.528	0.000	1.791	0.000
		Arginase	-1.942	0.000	-1.094	0.000	-1.625	0.000
		Nitric-oxide synthase	-		-1.387	0.060	-1.999	0.013
		S-adenosylmethionine synthetase	-1.607	0.000	-2.170	0.000	-2.069	0.000
		D-amino-acid oxidase	-		-1.528	0.000	-	
		Cystathionine beta-synthase	-1.482	0.000	-		-	
		Threonine 3-dehydrogenase	-2.223	0.000	-		-	
		4-hydroxyphenylpyruvate dioxygenase	-		-1.221	0.000	-1.120	0.000
		Asparagine synthase	-		1.313	0.000	-	
		Histidine decarboxylase	-		-1.057	0.009	-	
		Homocysteine s-methyltransferase	-		1.032	0.013	-	
	Immune responses	Tumor necrosis factor superfamily member 2	-1.453	0.180	-		-	
		Interleukin 17 receptor E	2.121	0.000	1.823	0.000	-	
		Transcription factor AP-1	-1.748	0.000	-2.746	0.000	-	
		Protein Fos B	-2.134	0.000	-5.088	0.000	-	
		Transcription factor jun-D	-1.367	0.000	-1.964	0.000	-	
		Component 4	-		-1.690	0.000	-	
		Interleukin 2 receptor beta	-		-1.357	0.022	-	
		Interleukin 4 receptor	-		1.911	0.007	-	
		Interleukin 11	-		1.907	0.000	1.926	0.000
Spleen	Amino acid metabolism	Nitric-oxide synthase	-1.017	0.000			-5.382	0.000
		Arginase	-1.204	0.000	-2.510	0.000	-1.902	0.000
		Alanine transaminase	-1.354	0.058	-8.308	0.000	-6.852	0.000
		Glycine dehydrogenase	-1.566	0.000	-1.302	0.000	-1.536	0.000
		Glutaminase	1.045	0.000	1.084	0.000	-	
		L-amino-acid oxidase	-		-1.860	0.015	-1.570	0.024
		L-serine ammonia-lyase	-		-		-1.056	0.016
		Threonine 3-dehydrogenase	-		-		-1.750	0.000
		L-threonine ammonia-lyase	-		-		-1.056	0.016
		Aspartoacylase	-		-		-1.045	0.000
		Hydroxyproline dehydrogenase	-		-		-2.188	0.000
		Phenylalanine-4-hydroxylase	-		-		-1.229	0.000
		Glycine N-methyltransferase	-		-		-1.170	0.000
		Glycine cleavage system H protein	-		-		-1.986	0.000
	Immune responses	Complement component 4	1.165	0.000	2.089	0.000	-	
		Complement component 3	1.967	0.000	1.366	0.000	-	
		Complement component 8 subunit gamma	1.128	0.000	1.266	0.000	-	
		Complement component 9	1.057	0.000	1.156	0.000	-	
		Tumor necrosis factor superfamily, member 2	-1.423	0.000	-		-3.846	
		Interleukin 6 signal transducer	-1.522	0.000	-		-1.635	0.000
		Toll-like receptor 4	-1.633	0.002	-		-1.594	0.000
		Interleukin 4 receptor	-1.212	0.000	-		-1.441	0.001
		Proto-oncogene protein c-fos	-1.481	0.035	-		-1.376	0.000
		P38 MAP kinase	-1.097	0.000	-		-2.622	0.031

Complement component 8 subunit alpha	-	1.241	0.000	0.000

Note: “-” represented the genes with |log2 Fold Change| < 1 or *P* adj > 0.05 were considered as no-significantly differentially expressed.

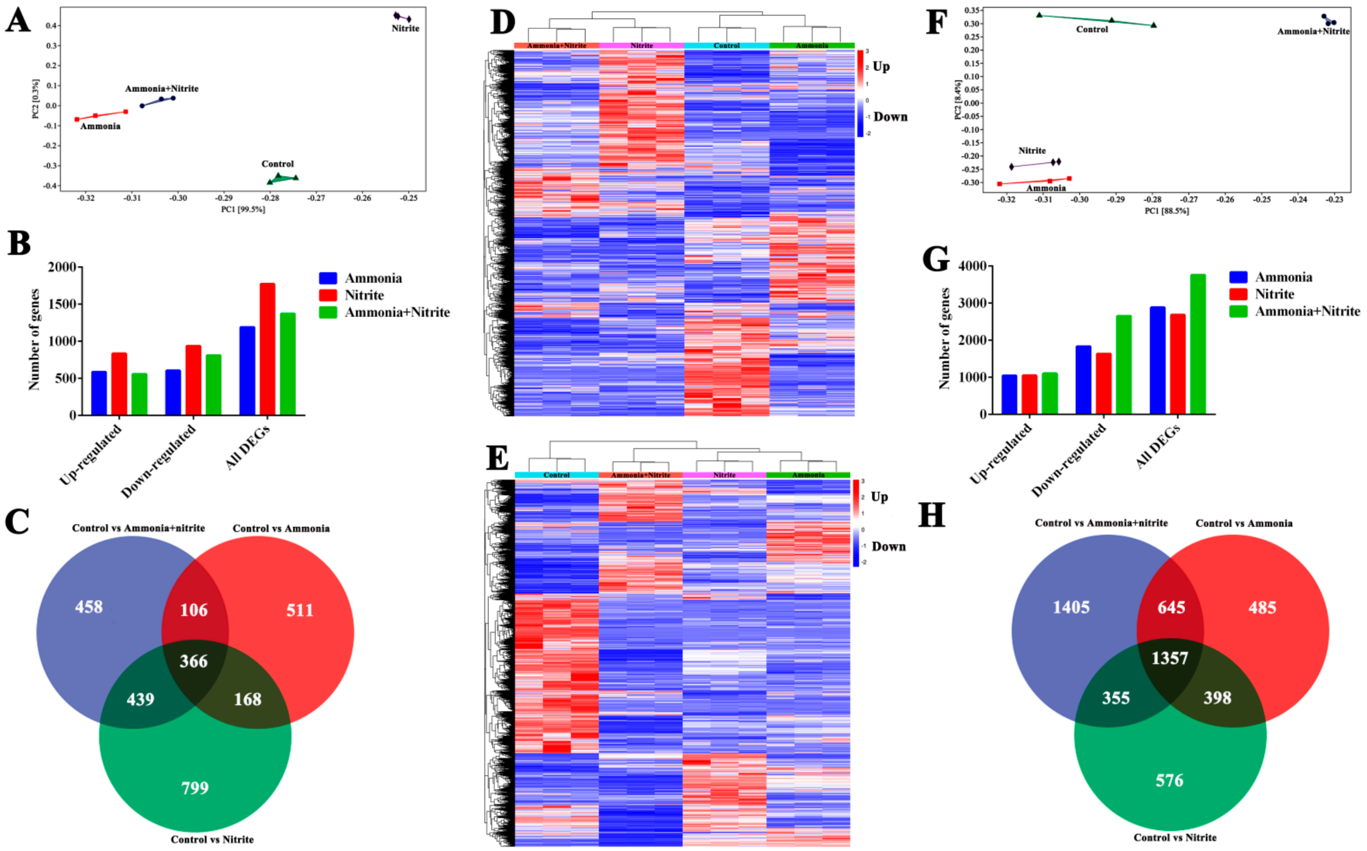

Figure S1. Transcriptomic analyse in silver carps after ammonia, nitrite, and their binary mixtures exposure for 48 h. (A: liver, F: spleen) Principal component analysis (PCA); (B: liver, G: spleen) Different numbers of DEGs; (C: liver, H: spleen) Venn diagram of DEGs; (D: liver, E: spleen) Hierarchical clustering for the differentially expressed genes.

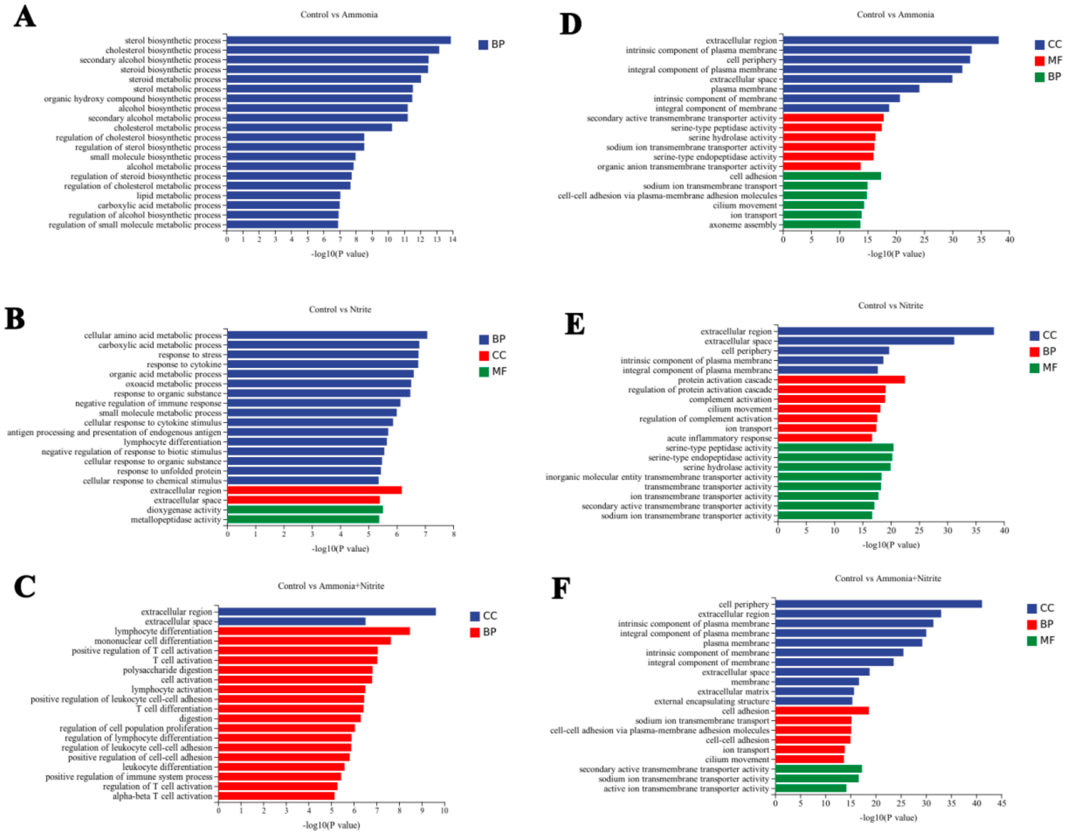

Figure S2. Go enrichment analysis bar chart (Liver: A-C; Spleen: D-F) CC: cellular component; BP: biology process; MF: molecular function.
